# The Impact of Promoter Variants in Interleukin-18 on Susceptibility to Ankylosing Spondylitis in a sample of Iraqi Patients

**DOI:** 10.12688/f1000research.172631.1

**Published:** 2025-12-15

**Authors:** Ibtehal Kadhim Jasim, Asmaa Mohammed Saud

**Affiliations:** 1 Biotechnology, University of Baghdad, Baghdad, Baghdad Governorate, Iraq

**Keywords:** Keywords: Ankylosing spondylitis, Interleukin-18, rs549908 Polymorphism, IL-18 serum levels, Genetic Susceptibilities.

## Abstract

**Background:**

Ankylosing spondylitis (AS) is a chronic inflammatory illness mainly influencing the axial skeleton. It is a multifactorial illness in which environmental and genetic parameters contribute to its cause, one of which is interleukins, including interleukin-18 (IL-18). Therefore, this study aimed to investigate the relations among single nucleotide polymorphism (SNP) rs549908 and serum levels of IL-18 in Iraqi patients with AS.

**Methods:**

During the period of November 2024 and January 2025, a total of 100 individuals were enrolled in the present work, including 50 patients with AS and 50 healthy controls (HC), were obtained from Baghdad Teaching Hospital. Using enzyme-linked immunosorbent assay (ELISA) and TaqMan real-time polymerase chain reaction (PCR), serum levels and rs549908 genotyping of IL-18 were estimated.

**Results:**

Significantly, levels of IL-18 in serum were raised in patients with AS in compared to HC (235.7060 *vs.* 151.10 pg/mL, p < 0.001). Genetically, T allele frequency (69.0% vs. 49.0%, *p =* 0.006) and TT genotype frequency (53.1% vs. 23.5%, *p =* 0.004) were significantly greater in patients with AS compared to HC. Analysis under the model of dominant (TG + GG *vs.* TT) demonstrated significant relations among status of illness and genotype (OR = 0.29, *p =* 0.007).

**Conclusions:**

These outcomes suggest rs549908 SNP and serum levels of IL-18, are related with raised susceptibilities to AS in the Iraqi population.

## Introduction

Ankylosing spondylitis (AS) is a chronic, inflammatory illness that largely impacts the axial joints, like the peripheral joints, sacroiliac joints, entheses and spine. ^1^ In AS, ankylosis, or the growth of new bone, causes long-term impairment, decreased mobility and spinal fusion. ^2^ It's still unclear what exactly causes AS. It has been proposed that the interplay among genetic and environmental factors serves a vital purpose. ^3, 4^ Proinflammatory cytokines such as TNF-alpha and other interleukins mediate a variety of inflammatory illnesses and are important modulators of inflammatory processes in the setting of AS. ^5^ Adaptive and innate immune responses are regulated by the pleiotropic, pro-inflammatory cytokine interleukin-18. ^6^ Interleukin-18 is primarily produced by neutrophils, dendritic cells, chondrocytes, keratinocytes, osteoblasts, and macrophages. It is a member of the interleukin 1 (IL-1) family. Through the caspase-1 proteolytic enzyme actions, the 23 kDa protein is transformed into the 18 kDa protein. ^7^ Depending on the inflammatory setting, IL-18 have an effective function in both Th1 and Th2 immune responses. Together with IL-12, IL-18 induces the formation of interferon-gamma, which strengthens the Th1-mediated immune response. When IL-12 is absent, IL-18 activates Th2 immune responses. ^8^ IL-18, as a pleiotropic and pro-inflammatory cytokine, can be generated in substantial amounts following infection and involved in innate and acquired immunity, inflammatory responses, and tumorigenesis. ^9^ Dysregulation of IL-18 has the potential to precipitate inflammatory or autoimmune pathologies that pertain to host defense mechanisms, oncogenesis, allergic reactions, immune responses, and arthritic conditions, among others. ^10^ Furthermore, IL-18 may serve as a pivotal relation among systemic inflammation and aberrant bone remodeling in arthritic conditions, by promoting osteoclast production and accelerating bone resorption. ^11^ It may have an effective function in the initiation of AS. With the advancement of molecular biological methodologies, single nucleotide polymorphisms (SNPs) within genes have increasingly become prevalent method for investigating illness susceptibilities. ^12^ Production of IL-18 protein is controlled via the *IL-18* gene. ^13^ The human *IL-18* gene, carried on chromosome 11q22.2–q22.3, comprises six exons and contains various single nucleotide polymorphisms (SNPs). Among these, the rs549908 polymorphism is the focus of the current study. ^14^ Several *IL-18* gene polymorphisms have been demonstrated to be related to raised IL-18 levels in immune-mediated disorders. ^15^ This study aimed to investigate the relations among IL-18 rs549908 and the concentrations of IL-18 in the Iraqi patients with AS, and to assess their potential functions as genetic and inflammatory indicators in this population.

## Methods

### Subjects

In the work, 50 patients diagnosed with AS and 50 HC were chosen and obtained from Baghdad Teaching Hospital, Medical City, Baghdad, Iraq, between November 2024 and January 2025. Information on lifestyles, particularly smoking habits, clinical diagnoses, and histories of administered therapy, was obtained from patients with AS. The AS diagnosis was confirmed in accordance with the criteria of spondylarthritis International Society (ASAS) 2009 classification for axial spondylarthritis. ^16^ Patients with AS measured their activity of illness through the Bath AS Disease activity index (BASDAI). ^17^

### Included criteria

The range of age of patients with AS was 20 to 63 years. The HC were age- and sex-matched to the cases, with no previous history of autoimmune or inflammatory disorders.

### Excluded criteria

Individuals younger than 20 years or older than 63 years were excluded. Patients or controls with comorbidities, as well as those with autoimmune or inflammatory diseases such as inflammatory bowel disease, psoriasis, or rheumatoid arthritis, were also excluded.

### Collection of blood samples

A volume of 3 mL of peripheral blood was obtained from each participant into 5 mL EDTA tubes for extraction of DNA. In addition, 3 mL of blood was collected into gel tubes and placed in centrifuge for 15 minutes at 3000 rpm. The resultant serum were aliquoted and preserved at −20°C till the time of immunological analysis.

### Immunological assay

Serum levels of IL-18 were measured using ELISA kit (FineTest, Wuhan Fine Biotech Co., Ltd., China; Cat. No. EH0011), in accordance with the manufacturer's instructions. Optical density was assessed at 450 nm with a microplate reader (HumaReader HS, Human GmbH, Germany).

### Genotyping determination

**DNA extraction**

From peripheral blood, genomic DNA was isolated and collected in EDTA-containing tubes utilizing the EasyPureBlood Genomic DNA Kit (TransGen Biotech, Beijing, China; Cat. No. EE121), depending to the manufacturer's guidelines. The purified DNA was eluted in 50 μL of elution buffer and stored at −20 °C until further analysis.

**SNP genotyping**

To amplify IL-18 SNPs (rs549908), TaqMan real-time polymerase chain reaction (RT-PCR) was utilised. Single reverse and single forward primers in addition to couple of probes were utilised and listed in Table 1. The primers and probes were custom-synthesized by Synbio Technologies (China). PCR mix (total volume: 20 μL) consisted of 5 μL nuclease-free water, 6 μL SuperMix, 1.5 μL forward primer, 1 μL of each probe, 4 μL DNA, and 1.5 μL reverse primer. The mix was transferred to a PCR program (TransGen Biotech SuperMix/China). Amplification was performed under standard conditions: an initial denaturation cycle 95°C for 2 min, followed by 30 cycles of of denaturation 95°C for 30 sec, annealing 58°C for 1 min and extension 72°C for 30 sec. Amplification products were additionally verified by 1.5% agarose gel electrophoresis.

**Table 1.** Primer and probe sequences used for genotyping IL-18 gene rs549908 SNP.

Name	Sequence (5′→3′)	Length (base)	Dye
Forward Primer	CTTATGACTGATAATTTAGATTCAAG	26	-
Reverse Primer	ATTGTAGCTACTTCTGGAACAG	23	-
Probe 1	TTGCCAAAGTAATCTGATTCCAGGTTTTCT	30	FAM
Probe 2	TTGCCAAAGTAATCGGATTCCAGGTTTTCT	30	HEX

FAM: Fluorescein amidite; HEX: Hexachlorofluorescein.

### Statistical analysis

Statistical analyses were carried out utilising SPSS software, version 20.0 (IBM Corp., Armonk, NY, USA). Descriptive statistics, including median, standard deviation, mean, percentage, and frequency were utilised to describe clinical and demographic data. The Shapiro-Wilk test was utilised for the distribution of continuous variables. Body mass index (BMI) and age followed a normal distribution and were compared among groups utilising the independent samples t-test, while erythrocyte sedimentation rate (ESR), Bath AS Disease activity index (BASDAI), and interleukin-18 (IL-18) levels displayed non-normal distributions and were examined utilising the Mann-Whitney U test. Frequencies of genotypic and allelic were estimated utilising the chi-square test, and deviations from Hardy-Weinberg equilibrium were also evaluated utilising chi-square analysis. 95% confidence intervals (CIs) and odds ratios (ORs) for relations of allele and genotype were detected utilising WinPepi software. Values p < 0.01 were regarded as extremely significant, and a p-value < 0.05 as statistically significant.

## Results

Based on the results in Table 2, the mean age of patients with AS was greater (39.9000 ± 11.23815 years) than that of the control group (31.0600 ± 10.46356 years), though the variation did not reach statistical significance (p = 0.059). Similarly, there was no significant variation in body mass index (BMI) among the AS group (28.2360 ± 5.18683 kg/m ^2^) and HC (26.7920 ± 3.80541 kg/m ^2^; p = 0.060). Sex distribution revealed a greater proportion of males among patients with AS (72%) compared to controls (60%), which was statistically significant (p < 0.001). A notable finding was the presence of a family history of AS in 22% of patients, while none of the controls reported a positive family history (p < 0.001), indicating a potential genetic predisposition in the patient group. Regarding inflammatory markers, erythrocyte sedimentation rate (ESR) was significantly elevated in patients with AS (18.62 ± 16.52937 mm/hr) compared to controls (8.28 ± 5.05092 mm/hr; p < 0.001), supporting an ongoing inflammatory state. C-reactive protein (CRP) was positive in 28% of patients with AS and negative in all control subjects (p < 0.001), further corroborating the presence of systemic inflammation in the AS group. Assessment of illness activity using the Bath Ankylosing Spondylitis Disease activity index (BASDAI) showed a mean score of 4.90 ± 1.00 in patients, while all healthy controls had a BASDAI of 0.00 (p < 0.001). Among patients with AS, 80% were classified as having moderate illness activity, and 20% had severe illness, whereas no illness activity was reported in the control group (p < 0.001).

**Table 2.** Demographic and clinical characteristics of AS and HC.

Parameter		AS (n = 50)	HC (n = 50)	*p-*value
**Age (mean ± S.D.) year**		39.9000 ± 11.23815	31.0600 ± 10.46356	0.059 NS
**Sex N (%)**	**Male**	36 (72%)	30 (60.0%)	<0.001 **
	**Female**	14 (28%)	20 (40.0%)	
**Family history N (%)**	**Yes**	11 (22%)	0 (0.0%)	<0.001 **
	**No**	39 (78%)	50 (100.0%)	
**Activity of illness N (%)**	**Moderate**	40 (80.0%)	0 (0.0%)	<0.001 **
	**Severe**	10 (20.0%)	0 (0.0%)	
**CRP N (%)**	**Negative**	36 (72%)	50 (100.0%)	<0.001 **
	**Positive**	14 (28%)	0 (0.0%)	
**ESR (Mean ± S.D.) mm/hr**		18.6200 ± 16.52937	8.2800 ± 5.05092	<0.001 **
**BMI (mean ± S. D.) kg/m** ^**2**^		28.2360 ± 5.18683	26.7920 ± 3.80541	0.060 NS
**BASDAI Mean ± S.D.**		4.90 ± 1.00	0.00 ± 0.000	<0.001 **

AS: Ankylosing spondylitis; HC: Healthy control; p: Probability; S. D.; Standard deviation; %: Percentage; Min: Minimum; max: Maximum; N: Number in each parameter; CRP: C-reactive protein; ESR: Erythrocyte sedimentation rate; BMI: Body mass index; BASDAI: Bath AS Disease activity index; NS: Non-significant.

** Highly significant.

As displayed in Table 3, serum IL-18 levels were significantly greater in patients with AS (median of 235.7060, range (23-485.78)) compared to HC (median 151.10, range (102.04-250.19)), with highly significant variations (p < 0.001).

**Table 3.** Interleukin-18 level of patients with AS in comparisons with HC.

Group	No. of subject	IL-18 (pg/ml) Median (min-max)	P-value
**AS**	50	235.7060 (23-485.78)	<0.001**
**HC**	50	151.10 (102.04-250.19)	

AS: Ankylosing spondylitis; HC: Healthy control; p: Probability; N: Number in each parameter; IL: Interleukin; pg/ml: Picograms per milliliter.

Table 4 illustrates the correlation analysis between serum IL-18 levels with clinical and inflammatory parameters in patients with ankylosing spondylitis. In particular, IL-18 revealed no significant correlation with BASDAI (p = 0.843), ESR (p = 0.915) or BMI (p = 0.884).

**Table 4.** Correlations of interleukin-18 levels with clinical and inflammatory parameters in patients with Ankylosing Spondylitis.

	BASDAI	ESR	IL-18	BMI
**BASDAI**	1	0.255	0.029	-0.086
**P-value **		0.074	0.843	0.553
**ESR**	0.255	1	-0.015	0.254
**P-value **	0.074		0.915	0.075
**IL-18**	0.029	-0.015	1	-0.021
**P-value **	0.843	0.915		0.884
**BMI**	-0.086	0.254	-0.021	1
**P-value **	0.553	0.075	0.884	

BASDAI: Bath ankylosing spondylitis disease activity index; Index; IL: Interleukin; P-value: Probability; ESR: Erythrocyte sedimentation rate; BMI: Body mass index.

**Figure 1.**
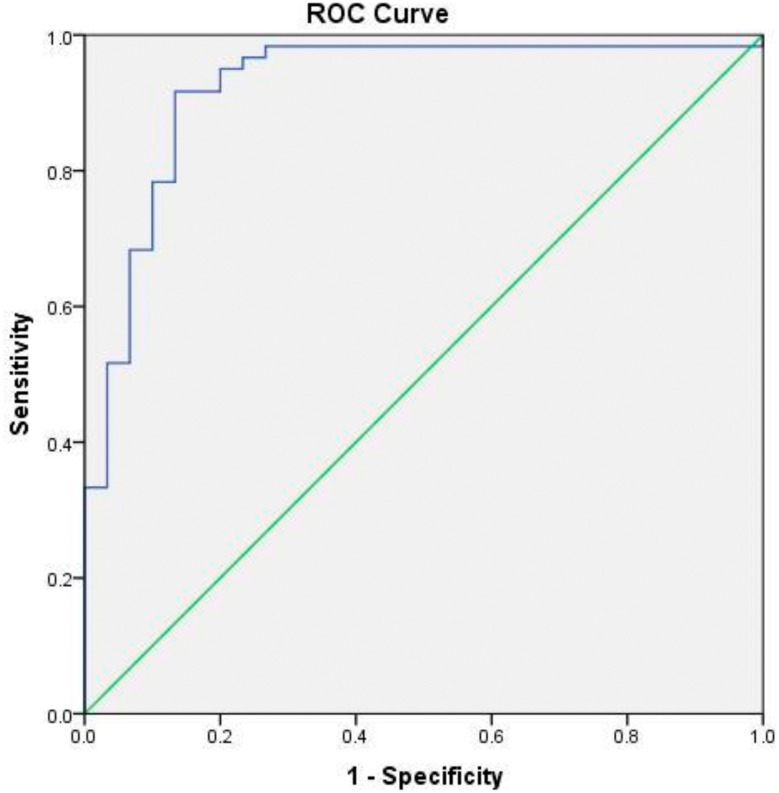
Receiver Operating Characteristic (ROC) curve of IL-18 for predicting susceptibility to ankylosing spondylitis.

To evaluate the diagnostic value of IL-18, a ROC curve was generated ( Figure 1). The analysis showed an AUC of 0.923 (95% CI: 0.859–0.988, p < 0.001), which reflected excellent diagnostic accuracy. These results suggested that IL-18 has strong potential as a biomarker for differentiating AS patients from healthy individuals.

Table 5, showed the frequencies of alleles and genotype of the IL-18 rs549908 polymorphism for both patients and HC. The results revealed that there was a significant relation among TT genotype and patients compared to HC (OR = 3.52, p *=* 0.004). In contrast, the TG and GG genotypes showed no statistically significant variations among groups (p *=* 0.15 and p *=* 0.21). The study found that the T allele was significantly more prevalent among patients with AS than in HC (OR = 2.32, p *=* 0.006). Conversely, G exhibited a relatively low frequency in patients, compared to HC (OR = 0.43, p *=* 0.006).

**Table 5.** Frequencies of alleles and genotype of the rs549908 of gene in AS and HC.

Genotype and Allele Frequencies	AS (n= 50)	HC (n= 50)	OR	95%CI	P-value
	N (%)	N (%)			
**TT**	26 (0.531)	12 (0.235)	3.52	1.51-8.19	0.004
**TG**	17 (0.347)	25 (0.490)	0.54	0.24-1.19	0.15
**GG**	7 (0.140)	13 (0.275)	0.46	0.17-1.27	0.21
**T**	69 (0.69)	49 (0.49)	2.32	1.36-4.27	0.006
**G**	31 (0.31)	51 (0.51)	0.43	0.24-0.77	0.006

AS: Ankylosing spondylitis; HC: Healthy control; OR: Odd ratio; C.I.: Confidence Interval.

**Figure 2.**
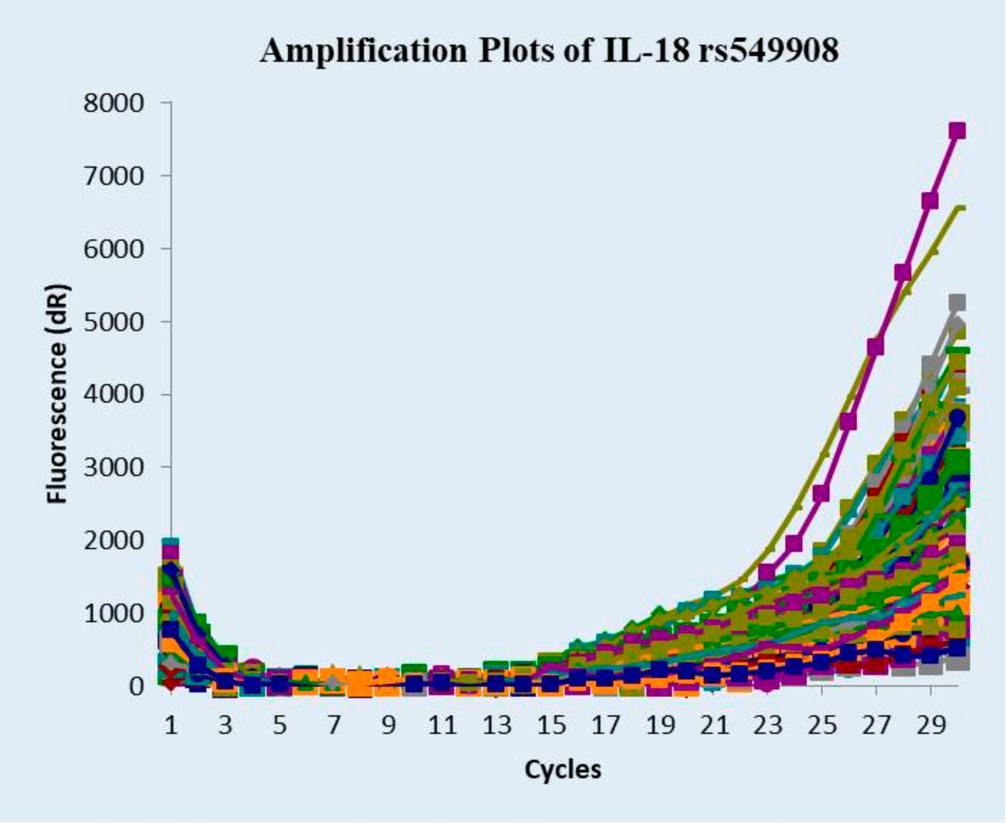
SNPs genotyping amplification plots of IL-18 rs549908.

The amplification plot illustrates the fluorescence intensity versus the PCR cycles for all samples in Figure 2. Each curve represents a single sample, showing successful amplification of IL-18 rs549908. The exponential phase was clearly visible after cycle 20, confirming the validity of the genotyping assay.

**Figure 3.**
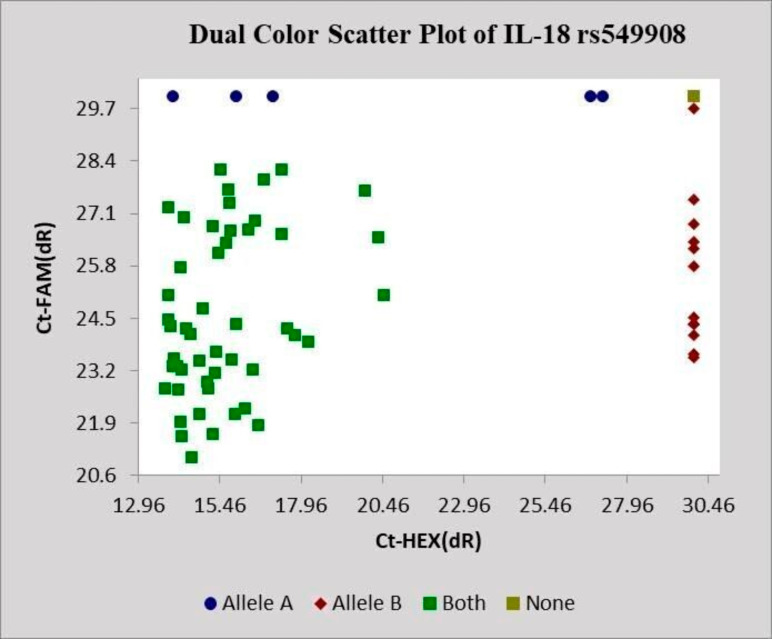
SNPs genotyping amplification plots showing fluorescence signals that indicate the alleles A and B in homozygous and heterozygous genotypes of IL-18 rs549908.

In Figure 3, the dual-color scatter plot displayed distinct separation of the genotypes. Samples were clearly distributed into three well-defined clusters corresponding to the TT, TG, and GG genotypes. Each point represented an individual sample, and the separation of the clusters provided clear discrimination between homozygous and heterozygous alleles.

To test Hardy-Weinberg equilibrium (HWE) for the IL-18 rs549908 polymorphism, a chi-squared test was conducted. As presented in Table 6, the genotype distribution in both the AS patient group (χ ^2^ = 2.10, p *=* 0.14) and the control group (χ ^2^ = 8.006, p *=* 0.99) showed no significant deviation from HWE. These outcomes demonstrated that genotype distributions were consistent with Hardy-Weinberg in both groups.

**Table 6.** Hardy-Weinberg equilibrium of the rs549908 of gene in the patients and HC.

HWE	AS (n = 50)		HC (n = 50)	
Genotypes	Observed	Expected	Observed	Expected
TT	26	23.8	12	12.0
TG	17	21.4	25	25.0
GG	7	4.8	13	13.0
X ^2^ value	2.10		8.006	
*p-*value	0.14		0.99	

AS: Ankylosing spondylitis; HC: Healthy control; HWE: Hardy-Weinberg equilibrium; O: Observed; E: Expected; p: Probability; χ ^2^: Chi-squared.

Based on the analysis of the genetic model provided in Table 7, the possible mode of inheritance of the polymorphism rs549908 regarding illness risk was assessed. In the dominant model, the combination of the TG and GG genotypes demonstrated a significant relationship with a protective effect against the illness (OR = 0.29, p *=* 0.007) in comparison to the TT genotype. On the contrary, the recessive model, which compared the GG genotype with the combined TG and TT genotypes, showed no statistically significant relation (OR = 0.46, p *=* 0.21). Likewise, the over-dominant model that compared TG genotypes with the combined TT and GG genotypes showed no significant relation (OR = 0.49, p *=* 0.10).

**Table 7.** Genetic model action of the *IL-18* gene.

Genetic model	Genotypes	Patients (n = 50)	HC (n = 50)	OR	95% C.I.	P-value
**Dominant**	**TG+GG**	17/7	25/13	0.29	0.13-0.68	0.007
	**TT (Ref.)**	26	12	1		
**Recessive**	**TG+TT (Ref.)**	17/26	25/12	1		
	**GG**	7	13	0.46	0.17-1.27	0.21
**Over-dominant **	**TT+GG (Ref.)**	26/7	12/13	1		
	**TG**	17	25	0.49	0.22-1.09	0.1

AS: Ankylosing spondylitis; HC: Healthy control; OR: Odd ratio; C.I.: Confidence Interval; P: Probability.

The relationship among IL-18 levels and rs549908 genotypes in both patients and HC with different illness activity was presented in Table 8. According to the outcomes, in the control group, IL-18 levels exhibited a significant level of consistency for different genotypes. In individuals with moderate illness activity, IL-18 concentration was elevated across all the genotypes, with GG (268.42 pg/mL), TT (239.06 pg/mL), and TG (222.26 pg/mL) genotypes having mean values that were greater when compared to the control group. Likewise, IL-18 was elevated in individuals with severe illness activity GG (251.52 pg/mL), TG (249.25 pg/mL), TT (231.03 pg/mL). There were statistically significant variations in the IL-18 levels in serum among the genotypes and activity of the illness (p *=* 0.001).

**Table 8.** Relationship among rs549908 genotypes and serum IL-18 levels according to illness activity in patients and HC.

Activity		rs549908	n	IL-18
**HC**		**GG**	**13**	153.218 (134.11-185.97)
		**TG**	**25**	142.7650 (131.00-175.10)
		**TT**	**12**	162.7315 (150.39-185.32)
**AS**	**Moderate**	**GG**	**5**	268.4210 (169.55-279.02)
		**TG**	**11**	222.2640 (187.09-391.88)
		**TT**	**23**	239.0620 (178.77-485.78)
	**severe**	**GG**	**2**	251.5230 (245.45-257.59)
		**TG**	**6**	249.2480 (205.66-333.46)
		**TT**	**3**	231.0290 (216.31-249.57)
***p-*value**				0.001

AS: Ankylosing spondylitis; HC: Healthy control; p: Probability; N: Number in each parameter; IL: Interleukin; pg/ml: Picograms per milliliter.

## Discussion

This study investigated the relation among the IL-18 rs549908 polymorphism and susceptibilities to ankylosing spondylitis (AS) in an Iraqi population by analyzing the distribution of genotypes and alleles, as well as evaluating serum IL-18 levels as a potential inflammatory biomarker. The IL-18 rs549908 polymorphism appeared to have a genetic component to since susceptibilities, since the investigation found significant variations in frequencies of alleles and genotype among patients with AS and healthy controls. Serum IL-18 levels were also noticeably greater in patients with AS, confirming the substance's function as an important inflammatory marker in the illness. ^18^ In this investigation, ankylosing spondylitis patients had considerably greater serum IL-18 concentrations (235.7060 pg/mL) than healthy controls (151.10 pg/mL; p = 0.001). Furthermore, the correlation between the serum IL-18 and clinical parameters, such as BASDAI, ESR, and BMI in the patients with AS, was examined and no statistically significant correlations were observed. These findings were consistent with previous reports, which indicated that although IL-18 levels were increased in AS patients, they did not significantly associate with clinical activity measures, suggesting that IL-18 may reflect disease susceptibility rather than activity. ^19^ Chromosome 11q22.2–22.3 contains the IL18 gene, and a number of variants in its promoter area have been linked to a raised risk of developing a number of inflammatory and autoimmune illnesses. ^15^ There was a strong correlation among the TT genotype and heightened vulnerability to ankylosing spondylitis (AS), according to the study of the IL-18 rs549908 genotype distribution. With an odds ratio (OR) of 3.52 (p = 0.004), 53.1% of patients with AS and 23.5% of healthy controls were found to have the TT genotype. On the other hand, the frequency of the G allele was 31.0% in individuals and 51.0% in controls (OR = 0.43, p = 0.006), suggesting a possible protective impact. In the Iraqi population under study, these results imply that the TT genotype could operate as a genetic predisposing factor for AS. A synonymous polymorphism found in the IL18 gene's exon region, the rs549908 variation had been linked in the past to a number of autoimmune disorders, such as atrophic conditions, systemic lupus erythematosus (SLE), and rheumatoid arthritis (RA). ^20^ In line with these outcomes, Abdulridha *et al.* found that immune-regulatory gene polymorphisms were substantially linked to a greater risk of autoimmune illnesses. ^21^ These results provide more credence to the theory that rs549908 played a role in the genetic vulnerability that underlies AS. Although this study focused specifically on the IL-18 rs549908 polymorphism. The outcomes aligned with broader research emphasizing the role of genetic variation in immune-regulatory pathways underlying autoimmune illness susceptibilities. Genetic model analysis indicated a significant dominant inheritance pattern, where individuals carrying TG or GG genotypes exhibited a markedly reduced risk of developing ankylosing spondylitis compared to TT homozygotes (OR = 0.29, p = 0.007). This suggested that the presence of the G allele might have conferred a protective effect against AS. Comparable relations were observed in studies of other immune-related disorders, where variants within immune-modulating genes were similarly linked to illness risk under dominant genetic models. These outcomes reinforced the notion that polymorphisms influencing immune function contributed to immune dysregulation and supported the multifactorial etiology of autoimmune illnesses. ^22, 23^ Serum IL-18 levels were further analyzed in relation to IL-18 rs549908 genotypes and illness activity scores in individuals with ankylosing spondylitis. Across all genotypes, patients with AS exhibited significantly greater IL-18 concentrations compared to healthy controls (p = 0.001). Among individuals with moderate illness activity, the highest serum IL-18 levels were observed in individuals with the GG genotype (268.42 pg/mL), followed by those with TT and TG genotypes. A similar trend was noted in patients with severe illness activity, although the absolute IL-18 values were slightly lower than in the moderate group. These outcomes suggest that while the TT genotype was associated with raised susceptibilities to AS, serum IL-18 levels did not correlate directly with genotype. This indicates that IL-18 levels may be regulated by additional factors beyond genetic variation at rs549908. Similar observations were reported by Doss *et al.*, who found no significant variations in serum IL-18 concentrations among different IL-18 genotypes in individuals with cutaneous lichen planus. ^24^ The markedly elevated serum IL-18 levels in AS patients, it was crucial to account for potential confounding factors such as biologic therapy, disease duration, and co-infections, which may affect IL-18 concentrations independently of genotype, demonstrating the need for caution in the interpretation of these elevated cytokine levels. The limited sample size within the GG subgroup, the immune system might vary among individuals and severity of illness which might have affected the cytokine levels rather than genotype. Although Limited sample size in our study reduces the ability to generalise study results, this remains the first study to correlate this polymorphism with AS development among Iraqi individuals. Additional research is necessary to examine rs549908 in autoimmune illnesses in Middle Eastern populations to improve regional comprehension of immune-related genetic variables. The present study demonstrated that specific IL-18 polymorphisms were significantly associated with reduced risk of ankylosing spondylitis (AS). The GT genotype, together with the corresponding protective allele, exhibited reduced susceptibility even after controlling for potential confounders. This observation aligns with findings from earlier research that have associated the genotypes GC (rs187238) and AG (rs360719) with a diminished risk of inflammatory disorders. These findings suggest a protective role of these IL-18 variants against AS, pointing toward a potential immunomodulatory mechanism whereby IL-18 genetic diversity influences inflammatory disease outcomes. This results align with and expand upon previous investigations of IL-18 polymorphisms in immune-mediated and inflammatory conditions. For example, Liang *et al.* reported associations of rs187238 and rs1946518 with biliary atresia, supporting the concept that IL-18 variants can alter disease risk in diverse contexts. ^25^ Similarly, Lando *et al* and Aboraia *et al.* identified rs549908 as a variant associated with total IgE levels in asthma patients across European populations, further reinforcing its involvement in immune dysregulation. ^26, 27^ The consistent implication of these polymorphisms across different inflammatory and immune-related conditions highlights their functional relevance. Interestingly, some studies reported divergent findings depending on population and disease phenotype, which may reflect genetic heterogeneity and environmental interactions. For instance, Eitan *et al.* found no association of rs187238, rs1946518, or rs549908 with alopecia areata in a Korean cohort, although associations were evident in Turkish populations. ^28^ Likewise, Mazurek-Mochol *et al.* observed no significant link between rs187238 and periodontitis risk but noted altered IL-18 expression and clinical periodontal measures among carriers of other genotypes. ^13^ These discrepancies emphasized the complex role of IL-18 as both a pro-inflammatory cytokine and, in some contexts, a potential protective factor. The findings were particularly notable because they suggested that IL-18 polymorphisms might have reduced the likelihood of AS. This contrasts with reports in other diseases where risk alleles contribute to pathology. This could indicate that in AS, specific allelic variants downregulate pro-inflammatory pathways or alter cytokine signaling in a manner that mitigates disease onset. Supporting this, de Almeida Viana *et al.* showed that rs187238 influenced lipid metabolism in COVID-19 patients, suggesting pleiotropic effects of IL-18 polymorphisms beyond classical inflammation. ^29^ Taken together, these findings imply that the biological effect of IL-18 variants may be disease and tissue-specific.

## Conclusion

This pilot study demonstrated that serum levels of IL-18 were significantly elevated in patients with ankylosing spondylitis (AS), suggesting a potential role of this cytokine in disease pathogenesis. Moreover, the T allele and TT genotype of the IL-18 rs549908 SNP were associated with an increased susceptibility to AS in the Iraqi population, whereas the G allele, particularly in the GG and TG genotypes, appeared to confer a protective effect under a dominant genetic model. However, these findings should be interpreted with caution. The relatively small study population limits the generalizability of the results, and the observed associations must therefore be considered preliminary and exploratory in nature. Larger studies across diverse populations are required to validate these findings and to clarify the functional impact of IL-18 gene polymorphisms in the onset and progression of AS.

## Ethical considerations

Ethical approval was obtained from the Biotechnology Department Ethics Committee at the College of Science, University of Baghdad (No. CSEC/0725/0086 on July 12, 2025). Every participant gave written informed consent before they enrolled.

## Data Availability

Zenodo. The Impact of Promoter Variants in Interleukin-18 on Susceptibility to Ankylosing Spondylitis in a sample of Iraqi Patients https://doi.org/10.5281/zenodo.17716124 (Ibtehal et al., 2025). ^30^ This project comprises the following underlying data:
• STROBE_checklist.pdf (Completed STROBE checklist for this study). • Raw_Data.xlsx (Individual raw data underlying all means, standard deviations, figures, and tables reported in the article). The data is available under the Creative Commons Attribution 4.0 International (CC-BY 4.0) license. The assay kits utilized in this investigation are commercially available. The manufacturer procedures are accessible via the following links:
• EasyPure ^®^ Blood Genomic DNA Kit (TransGen Biotech): https://www.transgenbiotech.com/genomic_dna_purification/easypure_blood_genomic_dna_kit.html
• Human IL-18 ELISA Kit (Cat. No. EH0011, FineTest): https://www.fn-test.com/product/eh0011 This observational study was conducted in accordance with the STROBE (Strengthening the Reporting of Observational Studies in Epidemiology) reporting guidelines.
