# Supplementary material for: The Impact of Promoter Variants in Interleukin-18 on Susceptibility to Ankylosing Spondylitis in a Sample of Iraqi Patients
Source: F1000Res. 2026 May 14;14:1399. Originally published 2025 Dec 15. [Version 2] doi: 10.12688/f1000research.172631.2 (PMC13133617; doi:10.12688/f1000research.172631.2)
Supplement: Peer review history

# Reviewer report: Reviewer response for version 2

**DOI:** 10.5256/f1000research.200795.r484857

**Published:** 2026-08-14

**Authors:** Raghad Hassan Hussein

**Affiliations:** 1 Department of Medical laboratory Techniques, College of health and medical techniques, Middle Technical University, Baghdad, Iraq

the language of the paper has been greatly improved

Is the work clearly and accurately presented and does it cite the current literature?

Yes

If applicable, is the statistical analysis and its interpretation appropriate?

Yes

Are all the source data underlying the results available to ensure full reproducibility?

Yes

Is the study design appropriate and is the work technically sound?

Yes

Are the conclusions drawn adequately supported by the results?

Yes

Are sufficient details of methods and analysis provided to allow replication by others?

Yes

Reviewer Expertise:

Immunity and inflammation, immunogenetics

I confirm that I have read this submission and believe that I have an appropriate level of expertise to confirm that it is of an acceptable scientific standard.

# Reviewer report: Reviewer response for version 1

**DOI:** 10.5256/f1000research.190371.r475867

**Published:** 2026-04-30

**Authors:** Sahar m Hussein

**Affiliations:** 1 Al-Nahrain University, Baghdad, Iraq

The study contributed suitable and methodical information on the correlation between IL-18 rs549908 polymorphism and serum IL-18 expression levels with susceptibility to ankylosing spondylitis in Iraqi subjects.

 The incorporation of genetic and immunological analysis is an evident strength and adds to the biological significance of the results. This study is well suited for its research objectives, and the methodologies used are appropriate and adequately described to ensure reproducibility.

       Results are clearly presented and show statistically significant differences that validate the proposed role of IL-18 in inflammatory and immune pathways. ROC analysis was found to also reinforce the possible diagnostic potential of IL-18. The study also adds, regionally, useful data that add to the known genetic vulnerability of ankylosing spondylitis, and to more generalized findings of this disease, particularly among underrepresented populations.

 A Few points for Improvement.

 1- Style: Language and scientific style: Some other language modifications could streamline the paper so that it is easier to read simply by removing the complex or repetitive structures.

 2- Statistical clarity: The statistical quality is ok, however, slight improvement in the consistency of the terms and reporting would provide greater clarity.

 3- ROC curve: This clinical association, such as it is in ROC, can be improved through diagnostic metrics (specific cut-off, sensitivity, and specificity) and these outcomes can also be integrated into that evaluation in the clinical literature.

 4- Discussion enhancement: A few studies of immune-related genetic polymorphisms among individuals with ankylosing spondylitis from different populations would slightly supplement the discussion.

    Although the reported findings are strong and well-described, future studies with larger cohorts and multivariate analytical techniques would be instrumental to successful generalizability, including advancing a broader perspective regarding the independent role of IL-18 genetic variants to disease susceptibility.

 Finally: The manuscript is scientifically sound, and the conclusions are supported by the presented data. The suggested points are minor and intended to further enhance clarity and presentation. The work is suitable for indexing.

Is the work clearly and accurately presented and does it cite the current literature?

Yes

If applicable, is the statistical analysis and its interpretation appropriate?

Yes

Are all the source data underlying the results available to ensure full reproducibility?

Yes

Is the study design appropriate and is the work technically sound?

Yes

Are the conclusions drawn adequately supported by the results?

Yes

Are sufficient details of methods and analysis provided to allow replication by others?

Yes

Reviewer Expertise:

molecular biology, molecular genetics , genetics , bioinformatics

I confirm that I have read this submission and believe that I have an appropriate level of expertise to confirm that it is of an acceptable scientific standard.

# Response

**Published:** 2026-05-01

**Authors:** Ibtehal kadhim

**Affiliations:** Biotechnology, University of Baghdad, Baghdad, Baghdad Governorate, Iraq

Dear Reviewer,

 We sincerely thank you for your constructive and encouraging comments.

 The manuscript has been revised to improve language clarity and reduce repetition. Statistical reporting has been refined for better consistency.

 The ROC analysis has been clarified, and the discussion has been strengthened by incorporating additional relevant studies.

 We believe these revisions have improved the manuscript.

 Sincerely,

 The Authors

# Reviewer report: Reviewer response for version 1

**DOI:** 10.5256/f1000research.190371.r475866

**Published:** 2026-04-25

**Authors:** Raghad Hassan Hussein

**Affiliations:** 1 Department of Medical laboratory Techniques, College of health and medical techniques, Middle Technical University, Baghdad, Iraq

This manuscript presents an  investigation into the association between the **I**L-18 rs549908 polymorphism and ankylosing spondylitis (AS) in an Iraqi population. The study addresses an important gap in the literature by providing population-specific genetic and immunological data, which are currently underrepresented in this field. The integration of molecular genetic analysis with serum cytokine quantification (ELISA) is a notable strength, offering a more comprehensive understanding of both genetic susceptibility and inflammatory status in AS. In conclusion, This study provides meaningful insights into the genetic and immunological factors underlying ankylosing spondylitis and represents a valuable contribution to the field, particularly in the context of Middle Eastern populations. .The manuscript would benefit from minor English editing to improve clarity and readability. For example:

- Replace “relations among” with “association between”
- Replace “illness activity” with “disease activity”

These are minor issues and do not affect scientific content.

Is the work clearly and accurately presented and does it cite the current literature?

Yes

If applicable, is the statistical analysis and its interpretation appropriate?

Yes

Are all the source data underlying the results available to ensure full reproducibility?

Yes

Is the study design appropriate and is the work technically sound?

Yes

Are the conclusions drawn adequately supported by the results?

Yes

Are sufficient details of methods and analysis provided to allow replication by others?

Yes

Reviewer Expertise:

Immunity and inflammation, immunogenetics

I confirm that I have read this submission and believe that I have an appropriate level of expertise to confirm that it is of an acceptable scientific standard.

# Response

**Published:** 2026-05-01

**Authors:** Ibtehal kadhim

**Affiliations:** Biotechnology, University of Baghdad, Baghdad, Baghdad Governorate, Iraq

Dear Reviewer,

 We thank you for your positive evaluation and helpful comments.

 The manuscript has been revised to improve clarity and readability. Terminology has been standardized throughout the text, including replacing “relations among” with “association between” and “illness activity” with “disease activity”.

 These revisions have improved the overall quality of the manuscript.

 Sincerely,

 The Authors

# Reviewer report: Reviewer response for version 1

**DOI:** 10.5256/f1000research.190371.r445404

**Published:** 2026-01-21

**Authors:** Duygu Kırkık

**Affiliations:** 1 Hamidiye Faculty of Medicine, University of Health Sciences, Istanbul, Turkey

This study presents a unique and regionally valuable contribution by addressing the association between the IL-18 rs549908 polymorphism and serum IL-18 levels with ankylosing spondylitis (AS) in an Iraqi population. The combined evaluation of genetic analysis and biochemical measurements is a strength of the study. However, as it stands, the article requires significant revision in terms of language, methodological clarity, statistical consistency, and interpretation.

 The text contains significant problems with English language and style.

 Frequently used expressions:

 “relations among” → “association between”

 “raised susceptibilities” → “increased susceptibility”

 “illness activity” → “disease activity”

 Many sentences are grammatically incorrect, long, and repetitive.

 Between the AS and control groups:

 Age difference was borderline significant (p = 0.059)

 Gender distribution was significantly different (p < 0.001)

 However:

 Logistic regression or multivariate analysis was not performed

 ORs were not adjusted.

 HWE results:

 For the control group, χ² = 8.006 and p = 0.99 are statistically inconsistent.

 Expressions such as “extremely significant” for p < 0.01 are not recommended in scientific writing.

 ROC analysis:

 AUC = 0.923 → very high

 However, cut-off, sensitivity, specificity, PPV/NPV are not provided

 The statistical analysis section should be restructured and the results simplified.

 The discussion could be further strengthened by incorporating recent population-based evidence supporting the role of innate immune–related genetic variants in ankylosing spondylitis. In line with the present findings, growing evidence suggests that genetic variations in immune signaling pathways contribute not only to disease susceptibility but also to clinical heterogeneity and disease activity in AS.

 Notably, **Kirkik et al.** demonstrated a significant association between the **TLR4 rs41426344 polymorphism and disease activity in Turkish patients with ankylosing spondylitis**, emphasizing the importance of immune-regulatory gene variants in AS pathogenesis across different populations ( *International Journal of Immunogenetics*, 2026) (Ref 1). Including this reference would provide valuable contextual support and reinforce the biological plausibility of the current genetic findings.

Is the work clearly and accurately presented and does it cite the current literature?

Yes

If applicable, is the statistical analysis and its interpretation appropriate?

Yes

Are all the source data underlying the results available to ensure full reproducibility?

Yes

Is the study design appropriate and is the work technically sound?

Partly

Are the conclusions drawn adequately supported by the results?

Yes

Are sufficient details of methods and analysis provided to allow replication by others?

Yes

Reviewer Expertise:

immunogenetics, anklosing spondilitis, polymorphisms, vaccine

I confirm that I have read this submission and believe that I have an appropriate level of expertise to confirm that it is of an acceptable scientific standard, however I have significant reservations, as outlined above.

## References

[1] : The Impact of TLR4 rs41426344 Polymorphism on Ankylosing Spondylitis Disease Activity: A Study in Turkish Patients. *International Journal of Immunogenetics* .2026;53(1) : 10.1111/iji.7002754-62 10.1111/iji.70027 41254827

# Response

**Published:** 2026-05-01

**Authors:** Ibtehal kadhim

**Affiliations:** Biotechnology, University of Baghdad, Baghdad, Baghdad Governorate, Iraq

Dear Reviewer,

 We sincerely thank you for your careful evaluation and valuable comments.

 The manuscript has been thoroughly revised to improve language clarity and style. Expressions such as “relations among” and “illness activity” have been corrected, and repetitive and complex sentences have been simplified.

 The statistical analysis section has been revised for clarity and consistency. The use of inappropriate terms such as “extremely significant” has been removed, and Hardy–Weinberg equilibrium calculations have been rechecked.

 The ROC analysis has been clarified to better reflect its diagnostic relevance.

 As suggested, the discussion has been strengthened by incorporating recent evidence on immune-related genetic variants, including the study by Kirkik et al. (2026).

 We believe these revisions have substantially improved the manuscript and addressed all concerns raised.

 Sincerely,

 The Authors
